# Glycogen Synthase Kinase 3 Inactivation Compensates for the Lack of CD28 in the Priming of CD8^+^ Cytotoxic T-Cells: Implications for anti-PD-1 Immunotherapy

**DOI:** 10.3389/fimmu.2017.01653

**Published:** 2017-12-11

**Authors:** Alison Taylor, Christopher E. Rudd

**Affiliations:** ^1^Leeds Institute of Cancer and Pathology (LICAP), University of Leeds, St James’s University Hospital, Leeds, United Kingdom; ^2^Division of Immunology-Oncology Research Center, Maisonneuve-Rosemont Hospital, Montreal, QC, Canada; ^3^Département de Médecine, Université de Montréal, Montreal, QC, Canada; ^4^Department of Pathology, Cell Signalling Section, Cambridge University, Cambridge, United Kingdom; ^5^Immune Venture Ltd., London, United Kingdom

**Keywords:** T-cells, glycogen synthase kinase-3, programmed cell death 1, Tbet, cancer

## Abstract

The rescue of exhausted CD8^+^ cytolytic T-cells (CTLs) by anti-Programmed Cell Death-1 (anti-PD-1) blockade has been found to require CD28 expression. At the same time, we have shown that the inactivation of the serine/threonine kinase glycogen synthase kinase (GSK)-3α/β with small-interfering RNAs (siRNAs) and small molecule inhibitors (SMIs) specifically down-regulates PD-1 expression for enhanced CD8^+^ CTL function and clearance of tumors and viral infections. Despite this, it has been unclear whether the GSK-3α/β pathway accounts for CD28 costimulation of CD8^+^ CTL function. In this article, we show that inactivation of GSK-3α/β through siRNA or by SMIs during priming can substitute CD28 co-stimulation in the potentiation of cytotoxic CD8^+^ CTL function against the EL-4 lymphoma cells expressing OVA peptide. The effect was seen using several structurally distinct GSK-3 SMIs and was accompanied by an increase in Lamp-1 and GZMB expression. Conversely, CD28 crosslinking obviated the need for GSK-3α/β inhibition in its enhancement of CTL function. Our findings support a model where GSK-3 is the central cosignal for CD28 priming of CD8^+^ CTLs in anti-PD-1 immunotherapy.

## Introduction

Naive T-cells are cells that have not encountered cognate antigen are essential for responses to novel pathogens. In this instance, activation requires a combination of stimulatory signals (1). The first signal is provided by the T-cell receptor (TCR) upon lymphocyte interaction with major histocompatibility class (MHC) antigens on the antigen-presenting cells (APCs) within the immune synapse (2). The second signal for T-cell activation is provided by CD28 and other costimulatory coreceptors on T-cells (3–6). CD28 is a well-defined costimulatory molecule found on lymphocytes, which interacts with B7 (CD80 and CD86) proteins on the APC (7, 8). TCR signaling alone can result in the lymphocyte undergoing cell death, or becoming anergic and thus unable to respond to antigen (9). Simultaneous signaling through CD28 and the TCR gives rise to sustained activation characterized by interleukin (IL)-2 production and cell-cycle entry (8, 10–12). Anti-CD28 crosslinking using monoclonal antibodies (MAbs) that augment CD28 cosignaling, especially on CD4^+^ T-cells, leading to increased interleukin 2-receptor (IL-2R), CD69 expression and proliferation (13). Conversely, Fab fragments of antibodies in mice, inhibit T-cell responses and can induce long-term heart allograft survival (14), and ameliorate experimental autoimmune encephalomyelitis (15).

We and others have shown that CD28 can complement and amplify TCR signaling (8, 12, 16, 17). In addition, CD28 can generate signals independently of TCR engagement (6, 18–20). The Tyr-Met-Asn-Met (YMNM) motif in the cytoplasmic tail of CD28 binds the adaptor growth factor receptor-bound protein 2 (GRB-2) (5, 11, 21–25) and the p85 regulatory subunit of phosphoinositide 3-kinase (PI-3K) resulting in the activation of AKT (21, 26, 27). This, in turn, leads to optimal *IL-2*-gene activation (11, 28), the expression of the anti-apoptotic protein BCL-X_L_, and the induction of an antigen response *in vivo* (11, 24, 29, 30). In this context, CD28 is linked to the serine threonine kinase; glycogen synthase kinase-3 (GSK-3). GSK-3 is constitutively active in T-cells, facilitating the exit of nuclear factor of activated T-cells (NFAT-c1) from the nucleus (31). CD28 signaling *via* PI-3K leads to the phosphorylation and inactivation of GSK-3, thus increasing IL-2 production and T-cell proliferation (32, 33).

Programmed cell death 1 (PD-1; PDCD1) is a member of the CD28 supergene family which negatively regulates T-cell function (3, 34, 35). PD-1 is expressed in response to T-cell activation and contributes to the exhaustion of CD8^+^ T-cells during chronic infection (36, 37). The coreceptor binds to ligands, programmed cell death ligand 1 and 2 (PD-L1/L2), on lymphoid and non-lymphoid cells (38–40). Immune checkpoint blockade (ICB) with anti-PD-1 or anti-PD-L1 has also proven highly successful in the treatment of human cancers, alone or in combination with anti-CTLA-4 (41, 42). PD-1 expression on tumor-infiltrating CD8^+^ T-cells correlates with impaired effector cell function (3, 43). We recently showed that GSK-3 is a central regulator of PD-1 expression and that the inactivation of GSK-3 using small molecule inhibitors (SMIs) downregulates PD-1 expression resulting in enhanced clearance of viral infections and cancer (44, 45). Recently, it has also been shown that PD-1 check-point blockade requires CD28 expression (46–48).

Here, we show that inhibition of GSK-3α/β by either small-interfering RNAs (siRNAs) or SMIs can substitute CD28 stimulation in the potentiation of CD8^+^ cytolytic T-cell (CTL) function. We propose that GSK-3 is the key mediator that is responsible for CD28 priming of CD8^+^ CTLs in T-cell immunity and in response to anti-PD-1 ICB immunotherapy.

## Results

**Figure 1:**
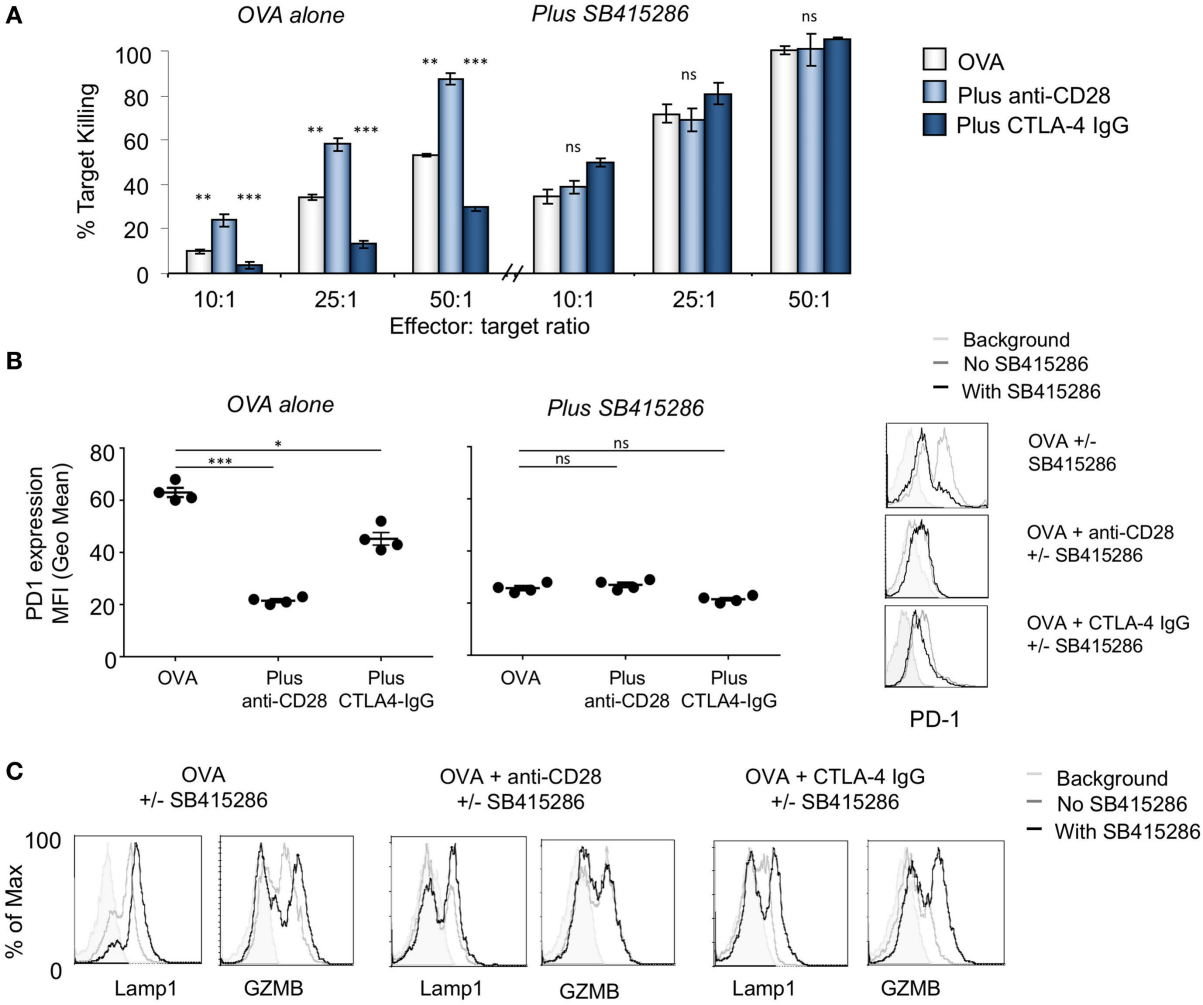
T-cell activation with anti-CD28 enhances cytolytic T-cell (CTL) killing of antigen specific target cells through glycogen synthase kinase 3 (GSK-3). **(A)** OT-1 CD8^+^ CTLs were activated with OVA peptide incubated in the presence (right panel) or absence (left panel) of SB415286 with or without anti-CD28 or blocking CD28 (CTLA-4 IgG fusion protein). After 5 days, CTLs were washed and counted before incubation with target (OVA-EL4) cells at the ratios shown for 4 h. Lactate dehydrogenase release was measured as an indication of target cell killing. Histogram depicts measurements normalized for background non-specific killing. OVA alone: light gray bars; anti-CD28: light blue bars; CTLA-4 IgG: dark blue bars (error bars based on triplicate values in individual experiments, data shown representative of four independent experiments). **(B)** Histogram showing MFI values of programmed cell death 1 (PD-1) expression as measured by flow cytometry. **(C)** Flow cytometry profiles of GZMB and Lamp-1 in the presence and absence of SB415286 alone, combined with anti-CD28 or CTLA-4 IgG. Error bars based on triplicate values in individual experiments; data shown representative of three independent experiments.

Recently, we reported that the inactivation of GSK-3α/β with siRNAs and drug inhibitors specifically downregulate PD-1 expression for enhanced CD8^+^ CTL function and clearance of tumors and viral infections (44, 45). We also previously reported CD28 costimulation can induce the phosphorylation of GSK-3 and hence its inactivation (33, 49). To assess CD8^+^ CTL function in response to antigen-presentation, we utilized MHC class I-restricted OVA specific-TCR transgenic (OT-1) mice with a TCR specific for the SIINFEKL peptide of OVAlbumin (OVA^257–264^) as presented by H-2k^b^. Control samples showed an increase in killing targets concurrent with an increase in effector/target (E/T) ratios. As previously shown (44), inhibition of GSK-3 with the SMI, SB415286, increased killing of EL4 target cells loaded with OVA peptide as measured at day 6 (Figure 1A). We next assessed the role of CD28 in this process. To this end, cultures were coincubated with soluble CTLA-4 IgG to block the interaction between CD28 and CD80/86 on presenting cells. EL4 cells express CD80 (50) and were therefore used as target cells. CTLA-4-IgG effectively inhibited the level of CTL killing of target cells (left panels). Intriguingly, the addition of SMI SB415286 completely restored normal levels of high CTL killing of targets at all E/T ratios (right panels). This ability of a GSK-3 SMI to bypass CD28 blockade by CTLA-4-IgG indicated that the inhibition of GSK-3 can substitute for the signal that is normally provided by anti-CD28. Further to this, as expected from our previous work, SB415286 suppressed the expression of PD-1 under all conditions (Figure 1B).

Anti-CD28 crosslinking has been found previously to augment CD28 signaling (13, 51). To assess this in the context of CD8^+^ CTLs, cultures were coincubated with anti-CD28 to crosslink the CD28 coreceptor for 7 days followed by an assessment of CTL function. Under these conditions, anti-CD28 greatly potentiated the killing potential of CTLs at all E/T ratios (left panel). Interesting, this level of enhanced killing was similar to that induced by GSK-3 SMI SB415286 (left panel). Further, the level of increased killing induced by anti-CD28 could not be further enhanced by SB415286 and *vice versa*. In the same vein, anti-CD28 coculture reduced the expression of PD-1 on CD8^+^ T-cells, similar to that seen with SB415286 (Figure 1B). Although it was originally assumed that CD28 would provide costimulation needed for the expression of PD-1 as in the case of CTLA-4 (52), we observed the opposite result. This was consistent with the generation of signals *via* GSK-3 whose inhibition also suppressed PD-1 expression. Consistent with this, CTLA-4-IgG blockade of CD28 was seen to increase PD-1 expression (left panel). This suggested that the normal engagement of CD28 by CD80/86 might also act to suppress PD-1 expression. Flow cytometry showed that SB415286 downregulated PD-1 expression on OVA peptide activated cells was accompanied by increased expression of Lamp-1 and GZMB in T-cells (Figure 1C).

**Figure 2:**
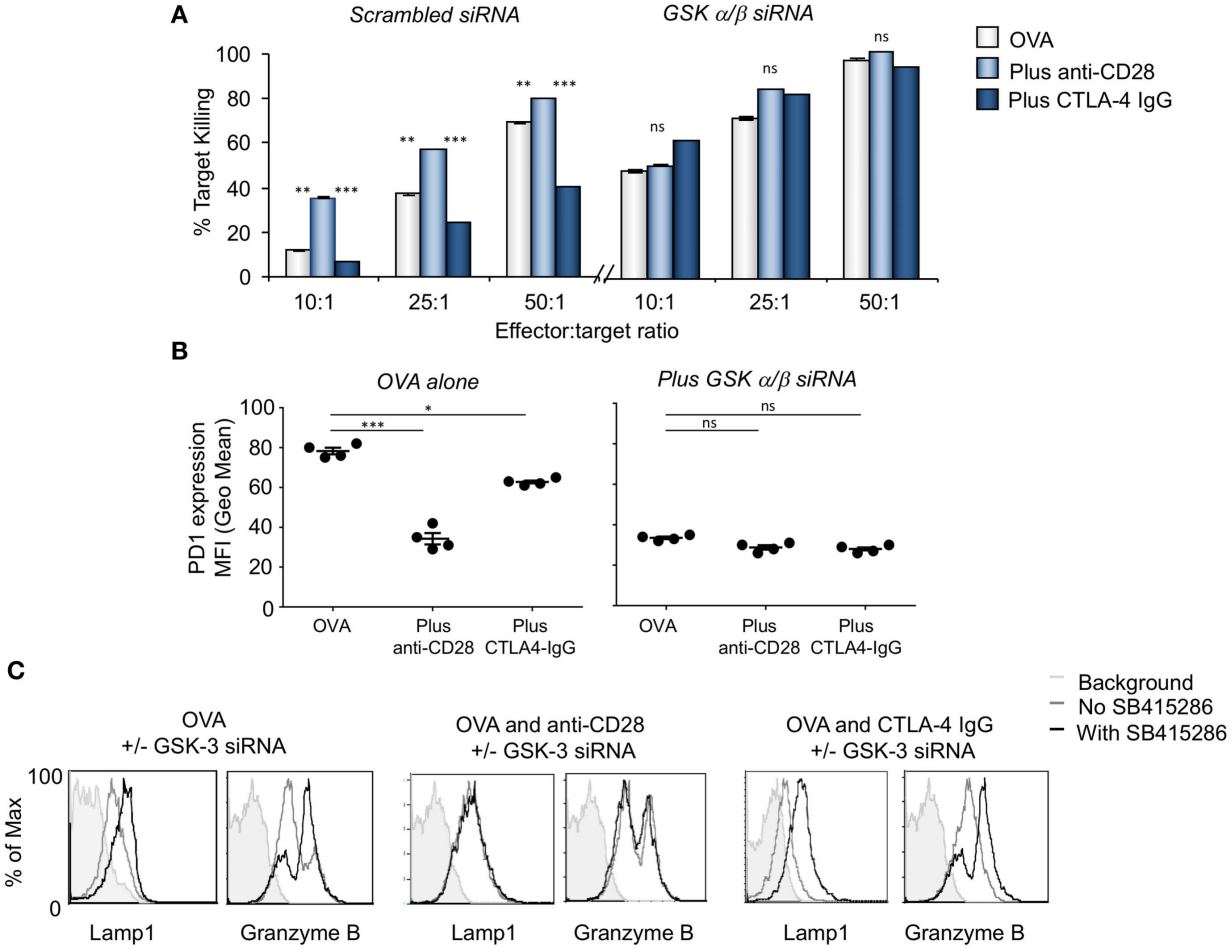
CD28 activation is comparable to glycogen synthase kinase 3 (GSK-3) inactivation enhancing cytolytic function. **(A)** OT-1 CD8^+^ cytolytic T-cells (CTLs) were transfected with scrambled (left panel) or GSK-3 (right panel) small-interfering RNA (siRNA) prior to activation with OVA peptide and incubated with or without anti-CD28 or blocking CD28 (CTLA-4 IgG fusion protein). After 5 days CTLs were washed and counted before incubation with target (OVA-EL4) cells at the ratios shown for 4 h. Lactate dehydrogenase release was measured as an indication of target cell killing. Histogram depicts measurements normalized for background non-specific killing. OVA alone: light gray bars; anti-CD28: light blue bars; CTLA-4 IgG: dark blue bars (error bars based on triplicate values in individual experiments, data shown representative of four independent experiments). **(B)** Histogram showing MFI values of programmed cell death 1 (PD-1) expression as measured by flow cytometry. **(C)** Flow cytometry profiles of GZMB and Lamp-1 in either scrambled or GSK-3 siRNA transfected cells stimulated with Ova alone, or combined with anti-CD28 or CTLA-4 IgG. Error bars based on triplicate values in individual experiments; data shown representative of three independent experiments.

In a related approach, anti-CD28 or CTLA-4 IgG was added to cells expressing siRNA for GSK-3α/β (Figure 2). In the scrambled control, anti-CD28 acted to increase the level of response. In addition, the knock-down of GSK-3α/β with siRNA increased the level of response to that of anti-CD28 such that the addition of anti-CD28 has no further effect. While CTLA-4-IgG markedly reduced the response of OT-1 T-cells expressing scrambled siRNA, it had no effect on cells expressing GSK-3α/β siRNA. Using a different approach, these data confirmed that GSK-3 inhibition could substitute for the signal provided by anti-CD28. In turn, the increased killing was reflected by a decrease in PD-1 expression (Figure 2B) and an increase in GZMB and Lamp-1 expression (Figure 2C).

**Figure 3:**
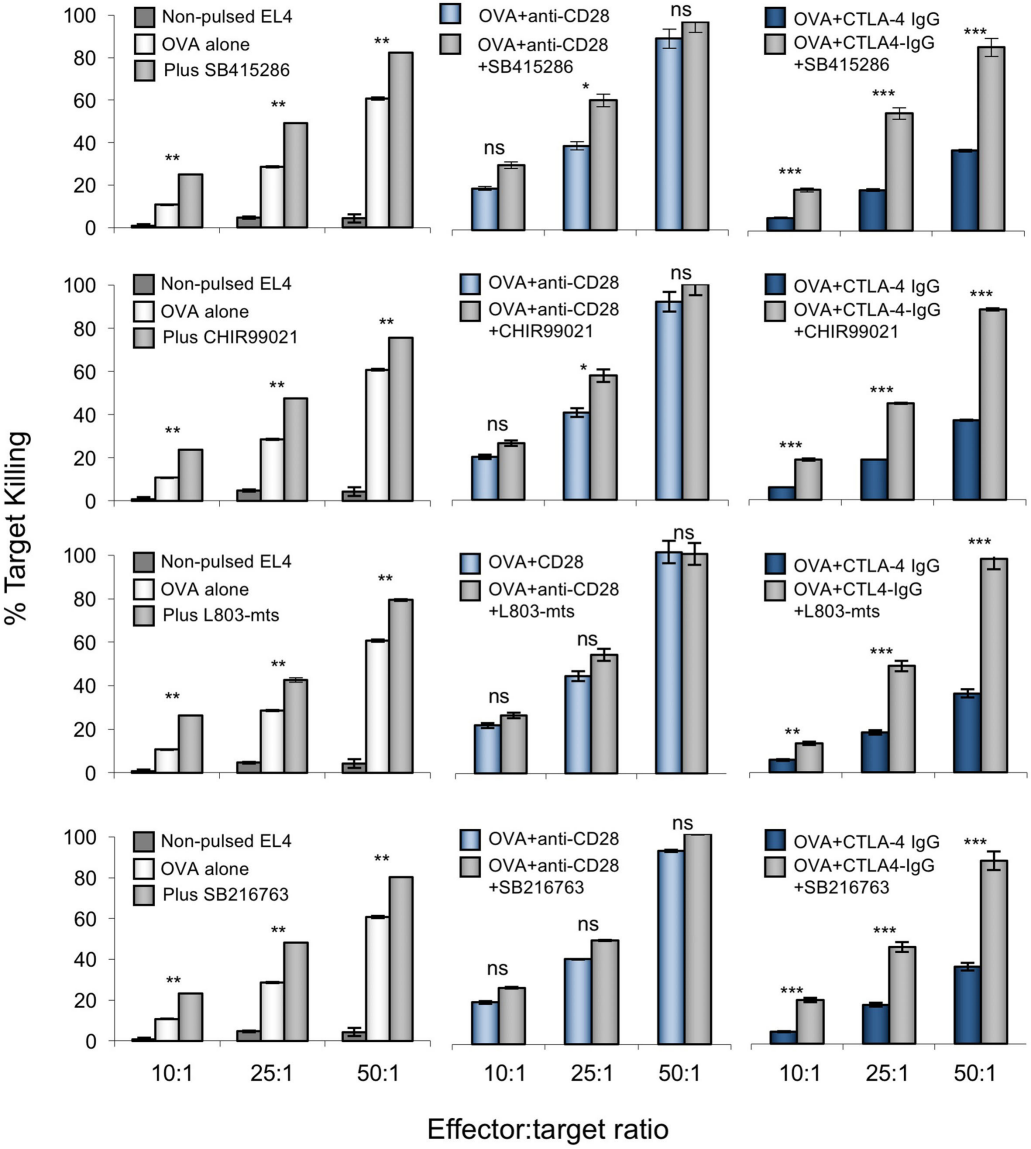
Anti-CD28 enhances cytolytic T-cell (CTL) killing of antigen-specific target cells to similar extent as glycogen synthase kinase 3 (GSK-3) inhibitors. OT-1 CD8^+^ CTLs were activated with OVA peptide incubated in the presence or absence of one of four small molecule inhibitors (from top to bottom; SB415286, CHIR99021, L803mts, SB216763) with or without anti-CD28 or blocking CD28 (CTLA-4 IgG fusion protein). After 5 days, CTLs were washed and counted before incubation with target (OVA-EL4) cells at the ratios shown for 4 h. Lactate dehydrogenase release was measured as an indication of target cell killing. Dark blue bars on left panel depicts background non-specific killing (non-pulsed target cell death). Error bars based on triplicate values in individual experiments; data shown representative of three independent experiments.

Importantly, the ability of GSK-3 inhibition to substitute for anti-CD28 in increasing CD8^+^ CTL function was seen with the use of different GSK-3 inhibitors; SB216763, CHIR99021, and L803-mts (Figure 3). Each have distinct structures but share a common target (53, 54). In each case, CD28 blockade by CTLA-4-IgG was reversed by the addition of any one of the four inhibitors used. Together, these data also support a key role for GSK-3 inhibition as a mediator of CD28 regulation of CD8^+^ T-cell killing.

**Figure 4:**
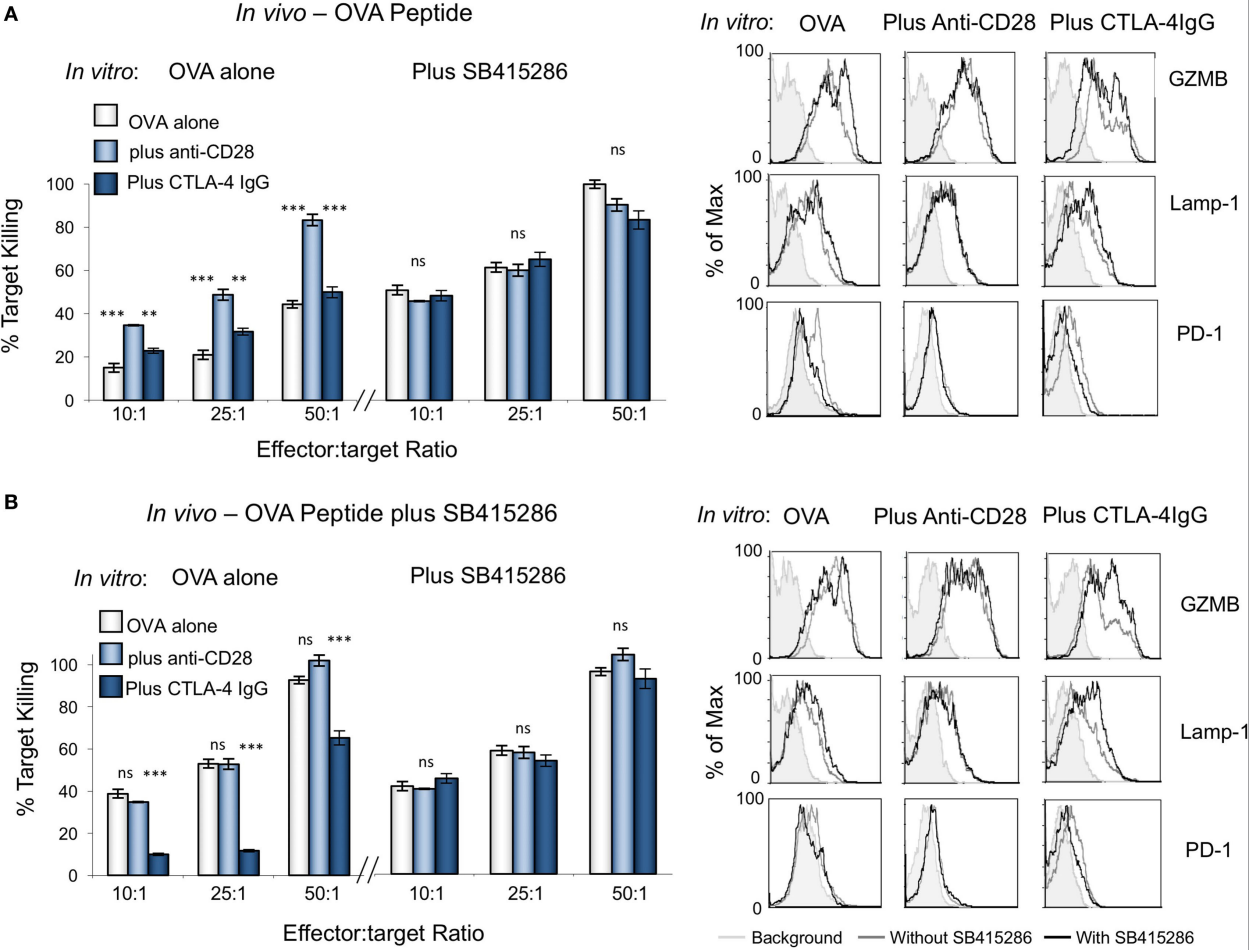
Cytolytic potential of cytolytic T-cells (CTLs) primed *in vivo* can be enhanced *in vitro* with anti-CD28 antibody. OT-1 mice were injected intravenously with ova peptide alone **(A)** or in combination with SB415286 **(B)**. Spleens were extracted on day 7*. Ex vivo* purified T-cells were then subjected to further stimulation with OVA peptide in the presence (right panel) or absence (left panel) of SB415286 with or without anti-CD28 or blocking CD28 (CTLA-4 IgG fusion protein). After 5 days, CTLs were washed and counted before incubation with target (OVA-EL4) cells at the ratios shown for 4 h. Lactate dehydrogenase release was measured as an indication of target cell killing. Histogram depicts measurements normalized for background non-specific killing. (Right panels) Flow cytometry profiles of GZMB, Lamp-1, and PD-1 in the presence and absence of SB415286, combined with anti-CD28 or CTLA-4 IgG (mean and SD of six mice per group). Error bars based on triplicate values in individual experiments; data shown representative of two independent experiments.

To assess the *in vivo* effect of CTL priming, OVA peptide in the presence or absence of SB415286 was injected intravenously into OT-1 transgenic mice followed by the harvest of spleens at day 7 (Figure 4). T-cells from extracted spleens were then subjected to further *ex vivo* stimulation for another 7 days in the presence or absence of SMI SB415286, anti-CD28, or CTLA-4-IgG followed by assessment of *ex vivo* killing of EL4-OVA targets. From this, it was observed that the *in vivo* administration of SMI enhanced cytolytic responses compared to OVA peptide alone (Figures 4A,B, left panel). This increase was also observed with OVA peptide alone primed cells when incubated with the GSK-3 SMI *in vitro* (Figure 4A, left panel). This finding showed that the cells were effectively primed *in vivo* with the SMI. In the case of cells primed with OVA peptide alone, the addition of anti-CD28 *in vitro* enhanced killing, whereas no additional effect was seen on cells primed with both OVA peptide and SMI. The addition of CTLA-4-IgG *in vitro* demonstrated the effects of priming with OVA peptide alone to be overcome by CD28 blockade. However, this was overcome by additional SMI *in vitro* (Figure 4B, left panel). Flow cytometry showed that priming with SMI, in addition to OVA peptide, slightly increased Lamp-1 and GZMB expression compared to OVA peptide alone. Further, anti-CD28 increased the numbers of CTLs expressing GZMB and Lamp-1, and this effect was reversed by CTLA-4-IgG (right panels). SMI had no further effect on anti-CD28-treated cells, but did overcome the CD28 blockade. Under both priming conditions, PD-1 expression was reduced in the presence of anti-CD28 to the same level as that seen with SMI. These data showed that GSK-3 inhibition *in vivo* augmented CTL function to a similar level as achieved *in vitro* with anti-CD28.

## Discussion

Both CD28 and the serine/threonine kinase GSK-3α/β have been found to play important roles in the activation of T-cells (4, 5, 44). The PI-3K/3-phosphoinositide-dependent protein kinase 1 (PDK1)/AKT signaling axis is central to cellular homeostasis, cell growth and proliferation (55, 56). We previously showed that GSK-3α/β inactivation with siRNAs and SMIs specifically downregulates PD-1 expression which leads to enhanced CD8^+^ CTL function and clearance of viral infections and cancer (44, 45). Despite this, it has been unclear how the GSK-3 pathway is linked to CD28 costimulation in the generation of CD8^+^ CTL function. We previously showed that CD28 has a cytoplasmic YMNM motif for binding to PI-3K, and that the pathway promotes the phosphorylation and inactivation of GSK-3 (21, 27, 33). The binding motif for PI-3K is phosphorylated by the src kinases, p56^lck^ and p59^fyn^ (22). Here, we show that GSK-3 inactivation substitutes for CD28 in the priming of cytotoxic CD8^+^ T-cells, while the enhanced cytotoxic function induced by anti-CD28 Mab crosslinking obviates the effects of GSK-3 SMIs.

Our first observation was that GSK-3 inactivation, using either siRNAs or SMIs, could substitute for CD28 in providing cosignals for enhanced cytotoxicity. GSK-3 inactivation reversed the effects of CD28 blockade with CTLA-4-IgG in the cytotoxic response OT-1 CTLs against EL4 cells expressing the OVA peptide. This was seen at all effector to target ratios studied. In each case this enhanced function was accompanied by an increase in Lamp-1 and GZMB expression. The efficacy of SMIs indicated that the inhibition of the catalytic activity of GSK-3, and not its potential role as a molecular scaffold for the binding of other proteins, was primarily responsible for increased function. Further, the effects were seen with four different SMIs with distinct structures whose shared property is the inhibition of GSK-3. These included ATP-competitive inhibitors SB216763, CHIR99021, and L803-mts, where SB216763 has a greater preference of inhibition for the GSK-3α isoform, while CHIR99021 and L803-mts preferentially inhibits GSK-3β (54, 57). Our previous work assessed longevity of the effectiveness of the SMIs by monitoring PD-1 expression in mice coinjected with EL4 tumors and a single injection of SMI. These data indicate that the effects of SB415286 were sustained for over 7–10 days (44).

The close relationship between CD28 and GSK-3 was also observed by the ability of anti-CD28 MAb crosslinking to over-ride or substitute for GSK-3 SMI inhibition in the potentiation of CTL function. While anti-CD28 blocks the interaction between CD28 and CD80/86, it also crosslinks the coreceptor in the generation of cosignals. CD28 crosslinking by CD80/86 is generally thought to be suboptimal, while the higher concentration of anti-CD28 can be more effective in occupying and crosslinking the coreceptor. Consistent with this, anti-CD28 MAb PVI greatly enhanced the killing function of OT-1 CTLs against OVA-EL4 targets. The level of increased killing was identical to the level observed with the addition of GSK-3 SMIs. The addition of GSK-3 SMI SB415286 to cultures that had been incubated with anti-CD28 provided no further potentiation of the CTL response and *vice versa*. This was confirmed in both *in vitro* and *in vivo* assays. This is reminiscent of the similarity in the effects of GSK-3 SMIs and anti-PD-1 blockade (44). Whether a similar relationship between GSK-3 and CD28 exists in CD4^+^ T-cells and operates in response to activating CD28 superagonists (58) remains to be studied.

**Figure 5:**
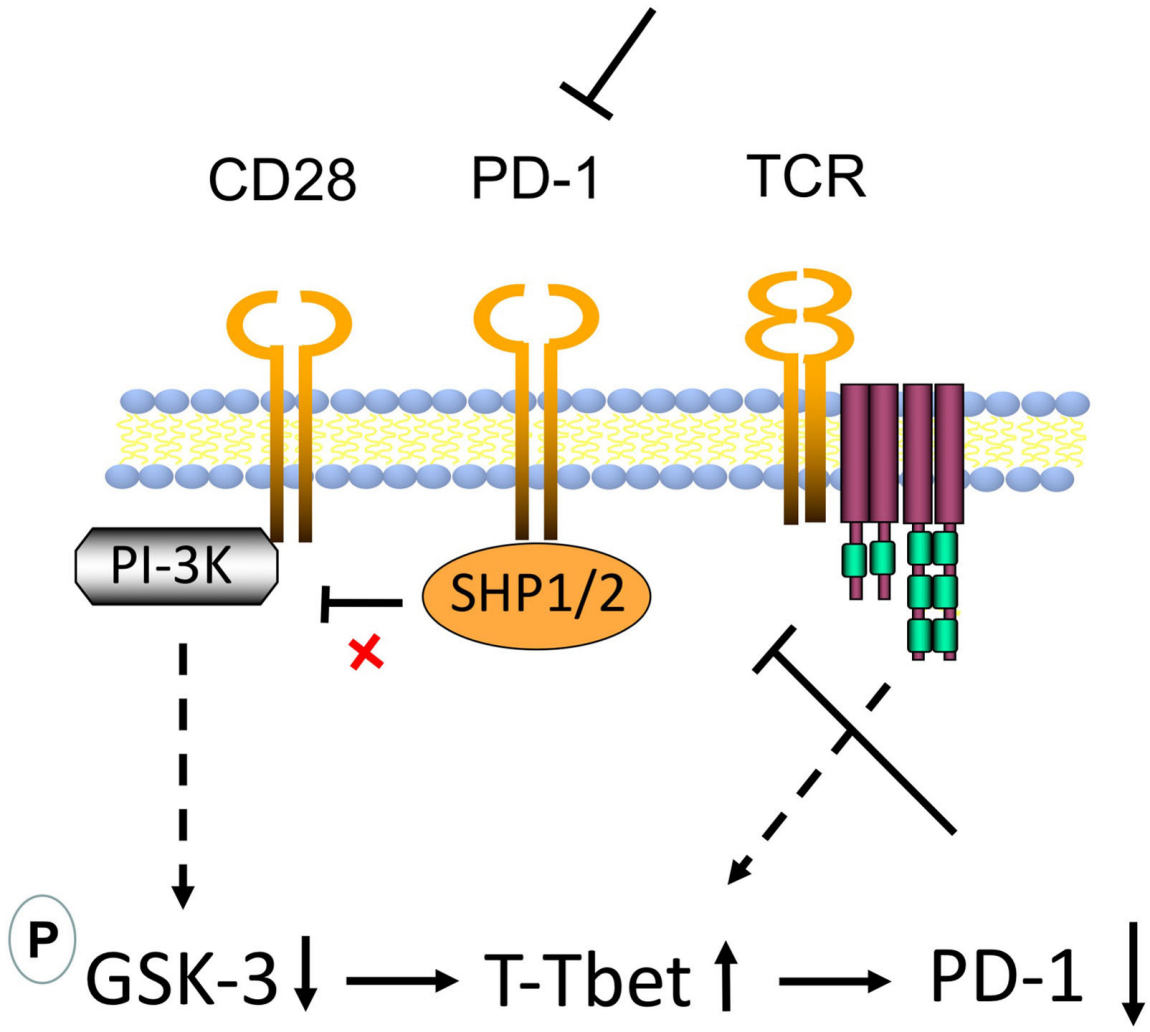
Model of CD28 mediated upregulation of CD8^+^ cytolytic T-cell (CTL) function *via* glycogen synthase kinase 3 (GSK-3) in the context of antiprogrammed cell death 1 (anti-PD-1) immune checkpoint blockade (ICB). In the absence of anti-PD-1 ICB, PD-1-associated phosphatases Src homology region 2 domain-containing phosphatase (SHP)-1 and SHP-2 dephosphorylate the CD28 phosphoinositide 3-kinase (PI-3K)-binding site Tyr-Met-Asn-Met (YMNM), thereby preventing the binding and engagement of PI-3K by CD28. In the presence of anti-PD-1, the activation of SHP-1/2 is blocked, allowing for the phosphorylation of the YMNM motif and the recruitment of PI-3K. PI-3K produces phosphatidylinositol (3,4,5) trisphosphates (PIP3) which serve as plasma membrane docking sites for proteins with pleckstrin-homology (PH) domains, including of the serine/threonine kinase AKT (also known as protein kinase B or PKB) and its upstream activator of the 3-phosphoinositide-dependent protein kinase 1 (PDK1). In our model, in T-cells, CD28 ligation by CD80/86 allows for the activation of PI-3K leading to the activation of PDK1 and the phosphorylation and activation of AKT. Phosphorylation of AKT at Ser473 by mTORC2 can also stimulate its full enzymatic activity. AKT in turn inhibits GSK-3 by phosphorylation [GSK-3α (Ser21) or GSK-3β (Ser9)]. We have shown that GSK-3 inhibition in turn upregulates the transcription of the transcription factor Tbx21 (Tbet) which in turn binds and inhibits transcription of PD-1. This pathway could downregulate PD-1 leading to more effective anti-PD-1 immunotherapy.

Overall, we propose a model where GSK-3 is the center of effects mediated *via* CD28 (Figure 5). Recently, it was reported that the rescue of exhausted CD8^+^ T-cells by anti-PD-1 blockade requires CD28 expression (46, 47). One proposed mechanism was the de-phosphorylation of CD28 by PD-1-associated Src homology region 2 domain-containing phosphatase (SHP)-2 (48). By connecting these observations to our findings, we propose a new model for the mechanism by which anti-PD-1 ICB operates in immunotherapy (see Figure 5). In the absence of anti-PD-1 ICB, PD-1-associated phosphatases SHP-1 and SHP-2 would dephosphorylate the CD28 YMNM motif for the activation of PI-3K. In the presence of anti-PD-1 ICB, the activation of SHP-1/2 is blocked, allowing for the phosphorylation of the CD28 YMNM motif and the recruitment of PI-3K (4, 5). PI-3K produces phosphatidylinositol (3,4,5) trisphosphates (PIP3) which serve as plasma membrane docking sites for proteins with pleckstrin-homology (PH) domains. CD28 induced PI-3K would promote PDK1 to the membrane where it would activate serine/threonine kinase AKT (also known as protein kinase B or PKB). AKT would in turn inhibit GSK-3 by phosphorylation of sites of human GSK-3α (Ser21) and GSK-3β (Ser9). As we have shown (44, 45), GSK-3 inhibition up-regulates the transcription of the transcription factor Tbx21 (Tbet) that inhibits PD-1 expression. We propose that CD28 regulation of GSK-3 accounts for the requirement for CD28 in the rescue of the response of CD8^+^ T-cells to anti-PD-1 blockade (46, 47). Further studies are needed to assess the full range of targets of the CD28-GSK-3-Tbet-PD-1 axis in T-cell biology.

## Materials and Methods

### Mice

C57BL/6–OT-1Tg and wt mice were used throughout the majority of the study. The research on mice was regulated under the Animals (Scientific Procedures) Act 1986 Amendment Regulations 2012 following ethical review by the University of Cambridge Animal Welfare and Ethical Review Body Home Office UK PPL No. 70/7544.

### Cells and Cultures

OVA specific CD8^+^ cytolytic T-cells were generated by incubating isolated splenocytes from OT-1 mice with SIINFEKL peptide of OVA (OVA_257–264_) at 10 ng/mL for 5–7 days. In certain cases, naive OT-1 T-cells were isolated from spleens using T-cell enrichment columns (R&D) and subjected to nuclear transfection (see method below). In the case of purified naive T-cells, the thymoma EL4 cell line was used to present OVA_257–264_ to primary T-cells. EL4 cells were incubated with 10 nM OVA_257–264_ peptide (Bachem) for 1 h at 37°C and treated with mitomycin C (Sigma-Aldrich, St. Louis, MO, USA) (final concentration of 10 µg/mL) prior to mixing with primary T-cells by coculturing at a ratio of 1:5 of EL4 and T-cells to generate cytotoxic T-cells. In either case, CTLs were generated in the presence or absence of SMI and/or anti-CD28 or CTLA-4-Ig (inhibitors/Abs added simultaneously with OVA-stimulation for 5–7 days) prior to washing and analysis by FACs, PCR, or cytotoxicity assays. Cells were cultured in RPMI 1640 medium supplemented with 10% FCS, 50 mM beta-mercaptoethanol, sodium pyruvate, 2 mM l-glutamine, 100 U/ml penicillin, and streptomycin (GIBCO).

### Antibodies/Reagents

Stimulations were performed using 10 nM OVA_257–264_ peptide (Bachem), anti-CD28 (clone PV1, bioXpress), and CTLA-4 IgG Fusion Protein (BD Pharmingen) where stated. SMI (GSK-3 inhibitor) was obtained from Abcam plc. and suspended in DMSO to give a stock solution of 25 mM and diluted to a concentration of 10 μM *in vitro*. Fluorescently labeled Abs to GZMB, PD-1, and Lamp-1 (CD107a) were obtained from Biolegend.

### Cytotoxicity Assays

Cytotoxicity was assayed using a Cytotox 96 nonradioactive kit (Promega) following the instructions provided. In brief, purified T-cells were plated in 96-well plates at the effector/target ratios shown using 10^4^ EL4 (ova peptide-pulsed) target cells per well in a final volume of 200 µl per well using RPMI lacking phenol red. Lactate dehydrogenase release was assayed after 4 h incubation at 37°C by removal of 50 µl supernatant from each well and incubation with substrate provided for 30 min and the absorbance read at 490 nm using the Thermomax plate reader (Molecular Devices). Percentage cytotoxicity = [(experimental effector_spontaneous_ − target spontaneous)/(target_maximum_ − target spontaneous)] × 100. All cytotoxicity assays were reproducible in at least three independent assays (59).

### Nuclear Transfection

The 3.0 μg GSK-3α/β siRNA was added to 1 × 10^6^ PBMC that had been washed in PBS and resuspended in 100 µl of Nucleofector™ solution for T-cells (Amaxa Biosystems, Cologne, Germany). Cells were transferred into a cuvette and electroporated using program X-01 of the Nucleofector™ (Amaxa Biosystems), and then immediately transferred into prewarmed cRPMI medium supplemented as recommended. GSK-3α/β specific and control siRNA were synthesized by Cell Signaling Technology. Control cells were transfected with 3.0 μg siRNA using the same protocol. Transfected cells were rested 24 h, before assays commenced.

### Priming OT-1Tg Cells *In Vivo*

Ova peptide (1 µg) was injected intravenously into OT-1Tg mice with and without SB415286 (100 µg) in 100 µl of PBS. Spleens were harvested after 7 days and T-cells purified before further stimulation *in vitro* for 5 days with the indicated antibodies.

### Statistical Analysis

The mean and SE of each treatment group were calculated for all experiments. The number of samples is indicated in the figure legends. Unpaired Student’s *t*-tests or ANOVA tests were performed using the InStat 3.0 software (GraphPad).*P < 0.05, **P < 0.01, and ***P < 0.001.

## Ethics Statement

The research was regulated under the Animals (Scientific Procedures) Act 1986 Amendment Regulations 2012 following ethical review by the University of Cambridge Animal Welfare and Ethical Review Body (AWERB) Home Office UK PPL No. 70/7544.

## Author Contributions

CR supervised, contributed conceptionally, and helped to write the article. AT contributed conceptionally, conducted experiments, and helped to write the article.

## Conflict of Interest Statement

The authors declare that the research was conducted in the absence of any commercial or financial relationships that could be construed as a potential conflict of interest.
